# Anti-Fungal Hevein-like Peptides Biosynthesized from Quinoa Cleavable Hololectins

**DOI:** 10.3390/molecules26195909

**Published:** 2021-09-29

**Authors:** Shining Loo, Stephanie V. Tay, Antony Kam, Fan Tang, Jing-Song Fan, Daiwen Yang, James P. Tam

**Affiliations:** 1 School of Biological Sciences, Nanyang Technological University, Singapore 637551, Singapore; snloo@ntu.edu.sg (S.L.); stay026@e.ntu.edu.sg (S.V.T.); k.antony@ntu.edu.sg (A.K.); fan003@e.ntu.edu.sg (F.T.); 2 Department of Biological Sciences, National University of Singapore, Singapore 117543, Singapore; dbsfjs@nus.edu.sg (J.-S.F.); dbsydw@nus.edu.sg (D.Y.)

**Keywords:** hevein-like peptide, hololectin, anti-fungal, quinoa, linker, chitin

## Abstract

Chitin-binding hevein-like peptides (CB-HLPs) belong to a family of cysteine-rich peptides that play important roles in plant stress and defense mechanisms. CB-HLPs are ribosomally synthesized peptides that are known to be bioprocessed from the following two types of three-domain CB-HLP precursor architectures: cargo-carrying and non-cargo-carrying. Here, we report the identification and characterization of chenotides biosynthesized from the third type of precursors, which are cleavable hololectins of the quinoa (*Chenopodium quinoa*) family. Chenotides are 6-Cys-CB-HLPs of 29–31 amino acids, which have a third type of precursor architecture that encompasses a canonical chitin-binding domain that is involved in chitin binding and anti-fungal activities. Microbroth dilution assays and microscopic analyses showed that chenotides are effective against phyto-pathogenic fungi in the micromolar range. Structure determination revealed that chenotides are cystine knotted and highly compact, which could confer resistance against heat and proteolytic degradation. Importantly, chenotides are connected by a novel 18-residue Gly/Ala-rich linker that is a target for bioprocessing by cathepsin-like endopeptidases. Taken together, our findings reveal that chenotides are a new family of CB-HLPs from quinoa that are synthesized as a single multi-modular unit and bioprocessed to yield individual mature CB-HLPs. Importantly, such precursors constitute a new family of cleavable hololectins. This unusual feature could increase the biosynthetic efficiency of anti-fungal CB-HLPs, to provide an evolutionary advantage for plant survival and reproduction.

## 1. Introduction

*Chenopodium quinoa* (*C. quinoa*), a pseudo-cereal from the Amaranthaceae family, has been grown as a staple in the Andean region for thousands of years. *C. quinoa* has high tolerance to extreme climate changes, such as drought, high salinity, and cold. Moreover, the high adaptability of *C. quinoa* promotes its survival and growth in marginal land at high altitudes (>3800 m above sea level). *C. quinoa* grains also have extensive nutritional value, and are a protein-rich food with a high vitamin and mineral content [1,2]. 

Nutritive plants, such as quinoa, can be threatened by plant pathogens. To defend against the pathogens, plants have evolved physical barriers and adaptive mechanisms to fight against, or establish a symbiotic relationship with, plant pathogens and pests [3,4,5]. An example is antimicrobial peptides produced by plants, which act as important effector molecules against plant microbial infections [6,7,8,9]. 

Plants produce a group of highly stable antimicrobial peptides that bind to chitin, a naturally occurring polysaccharide found in the exoskeleton of insects and the cell wall of fungi [10]. Hevein, the prototypic member of this group, is a 43 amino acid antimicrobial cysteine-rich peptide (CRP), discovered from the latex of the rubber tree (*Hevea brasiliensis*) [11,12,13]. The hevein sequence contains a chitin-binding (CB) domain and eight cysteine residues that are connected in a cystine knot [14,15]. The conserved CB domain (accession no. PS00026) is characterized by an SXφGφ (φ: aromatic amino acid residues are preferred for high-affinity binding) sequence in intercysteine loop 3, followed by GXXXXΦ in loop 4, where X represents any amino acid and Φ represents aromatic acid residues (Phe, Tyr, or Trp) [8] that play central roles in binding to the planar chitin monomers. 

Hevein belongs to the superfamily of plant CRPs, which are 2–6 kDa and have 6–10 Cys residues, with three to five disulfide bonds [8]. CRPs are classified into different families based on their cysteine content, cysteine spacing, and disulfide connectivity [8,16]. The multiple disulfide bridges confer stability against heat, acid, and protease-mediated degradation [17,18,19,20,21]. Of particular interest to our laboratory is a group of CRPs called hevein-like peptides (HLPs). Similarly to hevein, HLPs possess an evolutionarily conserved cysteine motif (CX_n_CX_n_CCX_n_CX_n_C) with a tandemly connecting CC motif at CysIII and CysIV [7,22,23,24,25,26,27,28,29,30,31]. HLPs can contain 6-, 8- or 10-Cys, and are further divided into two subfamilies, CB-HLPs and non-CB-HLPs (NCB-HLPs), depending on the presence or absence of a CB domain [16,20]. The CB domain determines the ability of CB-HLPs to bind to chitin, a biological signature for their anti-fungal activities.

CB-HLPs are ribosomally synthesized and post-translationally modified peptides [8,32]. The precursors of CB-HLPs are known to undergo an extensive post-translational process to release the mature peptides. They can be biosynthesized from a three-domained precursor, comprising of a signal peptide, mature domain, and a C-terminal tail [33]. However, CB-HLPs are also known to be derived from the lectin family. To date, only two biosynthetic pathways for the maturation of CB-HLPs from their lectin precursors have been reported [33]. According to their three-domain precursor architectures, the biosynthesis of CB-HLPs can be classified into the following two types: (1) type I non-cargo-carrying, and (2) type II cargo-carrying CB-HLPs [7,8,19,22,23,34]. The precursor of cargo-carrying CB-HLPs comprises a signal peptide, a mature CB-HLP, and a long C-terminal domain that contains a protein cargo, such as a class I chitinase-like or Barwin-like domain [33]. CB-HLPs without cargoes have short C-terminal domains of approximately 15–25 amino acids that have unknown functions [22,33]. The mature non-cargo-carrying CB-HLPs recognize chitin and have anti-fungal properties. 

Here, we report the isolation and characterization of a novel family of anti-fungal CB-HLPs, termed chenotides from quinoa. Different to the other known CB-HLPs, chenotides have a new type of cleavable tandem-repeating CB-HLP precursor architecture that could confer an evolutionary advantage for enhanced biosynthetic efficiency of anti-fungal peptides. Such an architecture constitutes the third type of biosynthetic precursors of CB-HLPs. 

## 2. Results and Discussions

### 2.1. Mass Spectrometry Screening of Quinoa Seed Extracts 

**Figure 1:**
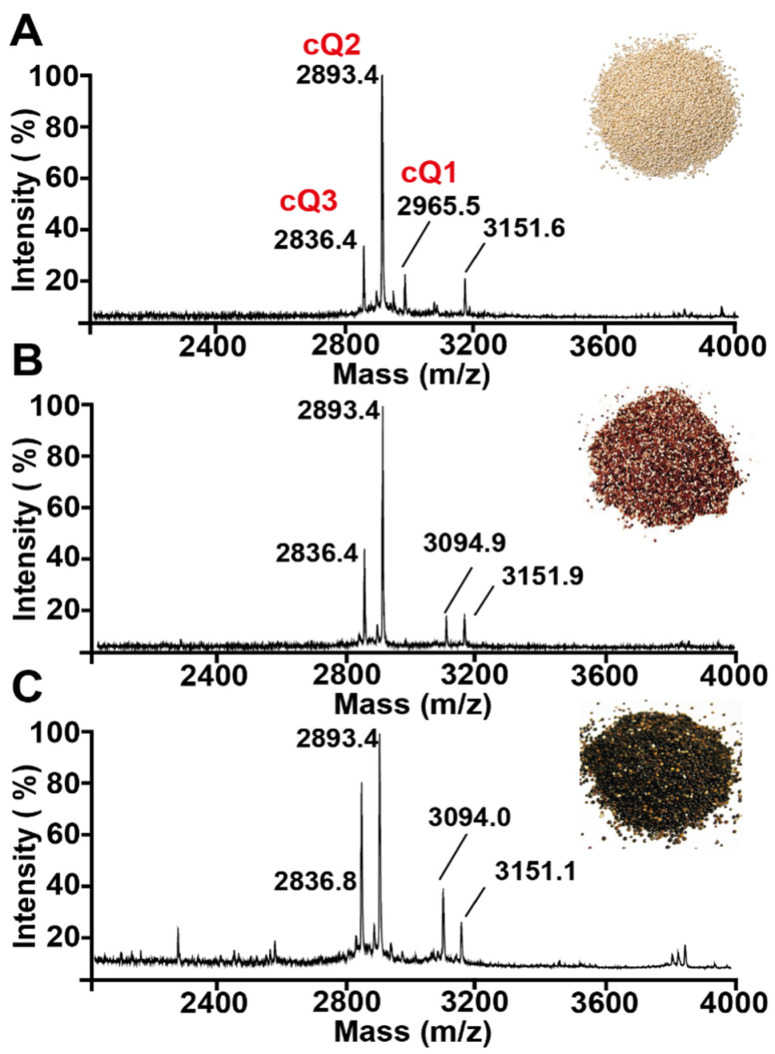
MALDI-TOF MS profile of aqueous quinoa extracts. (**A**) *C. quinoa* var. Willd, (**B**) *C. quinoa* var. red, (**C**) *C. quinoa* var. black. Clusters of peaks in the range of 2–4 kDa indicate the presence of putative cysteine-rich peptides. Chenotides cQ1, cQ2, and cQ3 are labeled with their corresponding peaks.

We used a mass spectrometry-driven approach to screen for CRPs in quinoa. The mass spectrometry profiles of aqueous extracts of *C. quinoa* var. Willd, *C. quinoa* var. red, and *C. quinoa* var. black seeds revealed clusters of putative CRPs between 2–4 kDa (Figure 1). To confirm that the identified peaks were CRPs, we performed *S*-reduction by dithiothreitol (DTT), and *S*-alkylation by iodoacetamide (IAM). For each successfully *S*-alkylated cysteine residue mediated by IAM, a 58 Da mass increase was be observed. The cysteine content was calculated based on the mass difference before and after *S*-reduction and *S*-alkylation. Our screening results showed that CRPs in quinoa contain six Cys residues (Supplementary Figure S1). 

### 2.2. Primary Sequence Determination of Chenotides

Scaled-up extraction was performed using 2 kg of *C. quinoa* var. Willd, and purified using strong cation exchange and C18 reverse-phased high-performance liquid chromatography. The CRPs isolated from *C. quinoa* var. Willd were designated as chenotides cQ1–3, and produced a yield of approximately 50 mg per kg of dried plant material. The chenotides cQ1–3 have molecular weights [M + H]^+^ of 2965 Da, 2893 Da, and 2836 Da, respectively.

**Figure 2:**
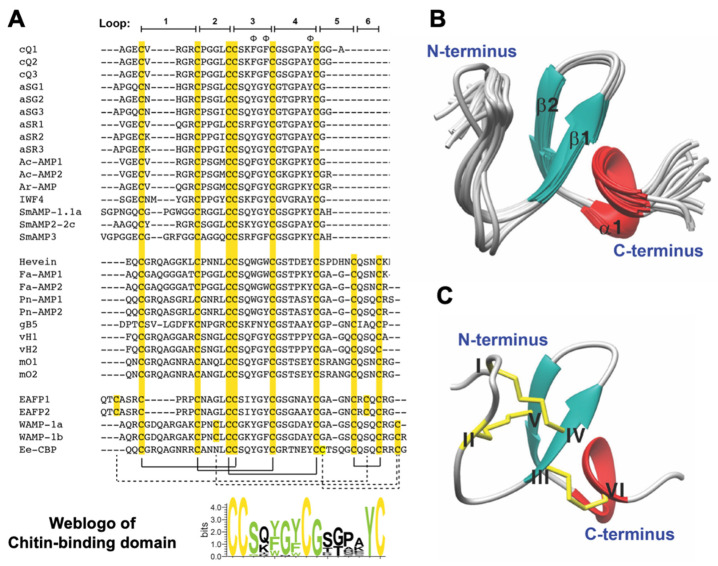
Sequence and solution structure of chenotide cQ2 (PDB:5ZV6). (**A**) Sequence alignment of chenotide cQ1, cQ2, and cQ3 with other reported chitin-binding hevein-like peptides, including altides aSG1–3, altides asR1–3, Ac-AMP1–2, Ar-AMP, IWF4, SmAMP-1.1a, SmAMP2-2c, SmAMP3, hevein, Fa-AMP1–2, Pn-AMP1–2, gingkotide gB5, vaccatide vH1–2, morintide mO1–2, EAFP1–2, WAMP-1a, WAMP-1b, and Ee-CBP. (**B**) Superposition of the chenotide cQ2 backbone traces from the final 20 ensembles solution structures and restrained energy minimized structure. (**C**) Ribbon representation of chenotide cQ2 structure. Φ represents aromatic residues essential for chitin-binding.

To determine the primary sequence of chenotides, the isolated peptides were first *S*-reduced with DTT and *S*-alkylated with IAM. The *S*-alkylated peptides were then subjected to enzymatic digestion with trypsin or chymotrypsin. The resulting peptide fragments were analyzed by MALDI-TOF MS and de novo sequenced using the b-ions and y-ions generated via MALDI-TOF MS/MS. Using chenotide cQ1 as an example, de novo peptide sequencing determined its primary sequence to be AGECVRGRCPGGLCCSKFGFCGSGPAYCGGA (Supplementary Figure S2). This sequence was confirmed against the cDNA sequence from GenBank. Our mass spectrometry results showed that chenotides cQ2 and cQ3 were N-terminal truncated sequences of cQ1 (Figure 2A, Table 1, and Supplementary Figures S3 and S4).

### 2.3. Solution Structure of Chenotide cQ2

The solution structure of chenotide cQ2 (PDB: 5ZV6) was determined using a total of 187 NMR-derived distance restraints and eight hydrogen bonds. Figure 2B shows the 20 lowest-energy structures of chenotide cQ2. The root mean square deviation (RMSD) value of the 20 best structures of chenotide cQ2 for the residues Glu3-Cys9 and Leu13-Try28 was 0.58 ± 0.24 Å, and for all the heavy atoms it was 1.21 ± 0.27 Å (Supplementary Tables S1 and S2). The chenotide cQ2 structure consists of two short extended anti-parallel beta strands (B1: Cys14-Ser16 and B2: Phe20-Gly22) and a one-turn alpha-helix (a1: Gly24-Try28) (Figure 2B). Similarly to other reported 6-Cys HLPs, chenotide cQ2 possesses a typical cystine-knot disulfide connectivity, which is as follows: CysI–CysIV, CysII–CysV, CysIII–CysVI (Figure 2C).

### 2.4. Peptide Stability of Cystine-knotted Chenotide cQ2

**Figure 3:**
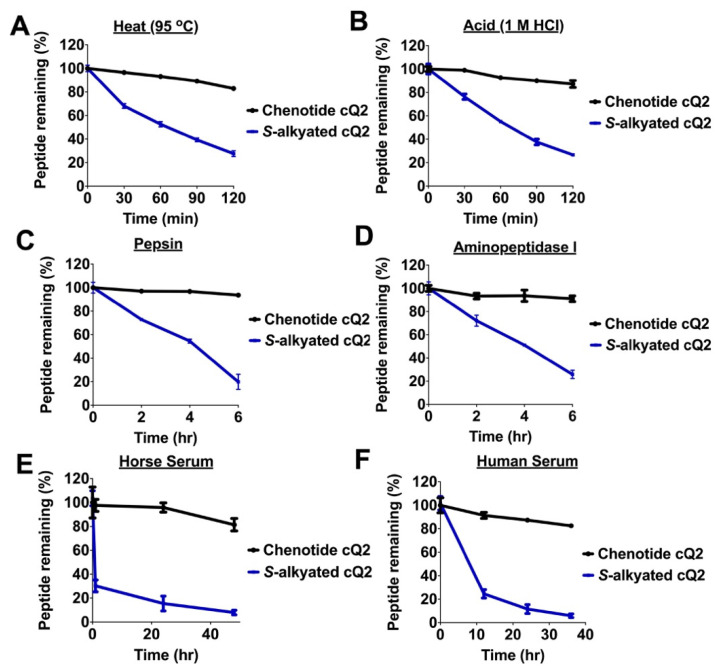
Chenotide cQ2 is hyperstable. Stability comparison of chenotide cQ2 and *S*-alkylated cQ2 (iodoacetamido-) under (**A**) heat (95 °C), (**B**) acid (1M HCl), (**C**) pepsin, (**D**) aminopeptidase I, (**E**) horse serum, and (**F**) human serum treatment as analyzed by RP-HPLC (*n* = 3).

Cysteine-rich peptides that are cross-linked by multiple disulfides are known for their stability against heat, acid, and proteolytic degradation [17,18,19,20,21]. Our results showed that chenotide cQ2 is indeed highly stable against heat (95 °C), acid (1M HCl), and proteolytic degradation (pepsin, aminopeptidase I, horse serum, and human serum) (Figure 3). In all the conditions, >80% of the peptides were retained after treatment, as monitored by RP-HPLC and MALDI-TOF MS. In contrast, *S*-alkylated chenotide cQ2 was not stable under the same conditions, indicating that the cystine-knot scaffold is responsible for the hyperstability of chenotides. 

### 2.5. Sequence and Structural Comparison of Chenotides with Other Chitin-Binding Hevein-Like Peptides

Our recent studies showed that many plant-derived CRPs contain a common cysteine motif (CX_n_CX_n_CCX_n_CX_n_C), with tandemly connecting cysteines in the third and fourth positions, which are arranged in a cystine knot [7,17,18,19,20,21,22,23]. This structural motif was first identified in hevein, an 8-Cys-CRP derived from the rubber tree, which has anti-fungal activities [11,12,13,14,15]. Hence, we termed CRPs that have this structural motif as HLPs. HLPs are grouped according to the number of Cys residues (6-, 8- or 10-Cys) in their primary sequence [8,16,20]. They are then further divided into two subfamilies, based on the presence or absence of a chitin-binding domain [8,16,20]. CB-HLPs are characterized by the presence of a chitin-binding domain that contains SXXG in intercysteine loop 3, followed by GXXXXΦ in loop 4, where X represents any amino acid and Φ represents an aromatic acid residue (Phe, Tyr, or Trp) [8,23]. Hevein is the prototypic member of 8-Cys CB-HLPs, and has a cysteine motif arranged as CX_8_CX_4_CCX_6_CX_6_CX_5_CX_3_C, cystine-knot disulfide connectivity, and a chitin-binding domain [11,12,13,14,15]. Non-chitin-binding HLPs (NCB-HLPs), on the other hand, have a similar cysteine motif to CB-HLPs, but lack the CB domain. Examples of NCB-HLPs, discovered by our laboratory, include roseltides [17,21,35], bleogens [16,18], and ginsentides [20].

**Figure 4:**
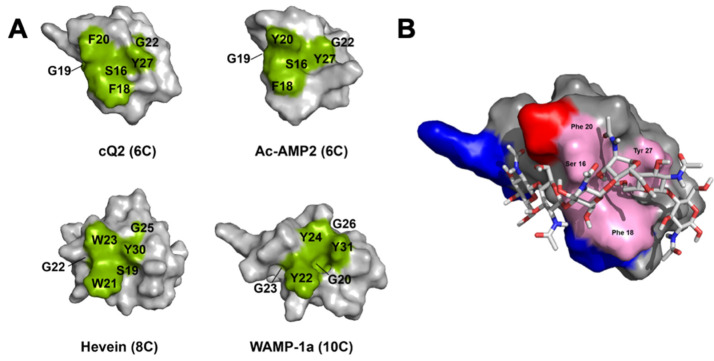
Chenotides belong to the family of chitin-binding hevein-like peptides. (**A**) Surface topology comparison of chenotide cQ2 (PDB: 5ZV6), Ac-AMP2 (PDB: 1MMC), hevein (PDB: 1HEV), and WAMP-1a (PDB: 2LB7). The residues highlighted in green represent the chitin-binding domain. (**B**) Surface representation of the peptide–ligand interaction between chenotide cQ2 and chitin (*N*-Acetylglucosamine hexamer). *N*-Acetylglucosamine is shown in ball and stick. Residues highlighted in blue and red are basic (Arg, His, Lys) and acidic (Asp, Glu), respectively.

BLAST analysis suggests that chenotides belong to the family of CB-HLPs, based on the presence of a chitin-binding domain. Sequence comparison and WebLogo analysis of chenotides with other known 6-, 8-, 10-Cys CB-HLPs revealed that the chitin-binding domain is highly conserved (Table 1 and Figure 2A). The surface topography of the chenotide cQ2 CB domain (Ser16, Phe18, Phe 20, and Tyr27) is similar to Ac-AMP2 [36], hevein [14,15], and WAMP-1a [37,38] (Figure 4A). The peptide–ligand interaction of chenotide cQ2 with an *N*-acetylglucosamine hexamer was modeled, and shows that chitin binds to the chitin-binding pocket of chenotide cQ2 through van der Waals and ch/π interactions (Figure 4B). 

### 2.6. Chenotide cQ2 Is Chitin-Binding

**Figure 5:**
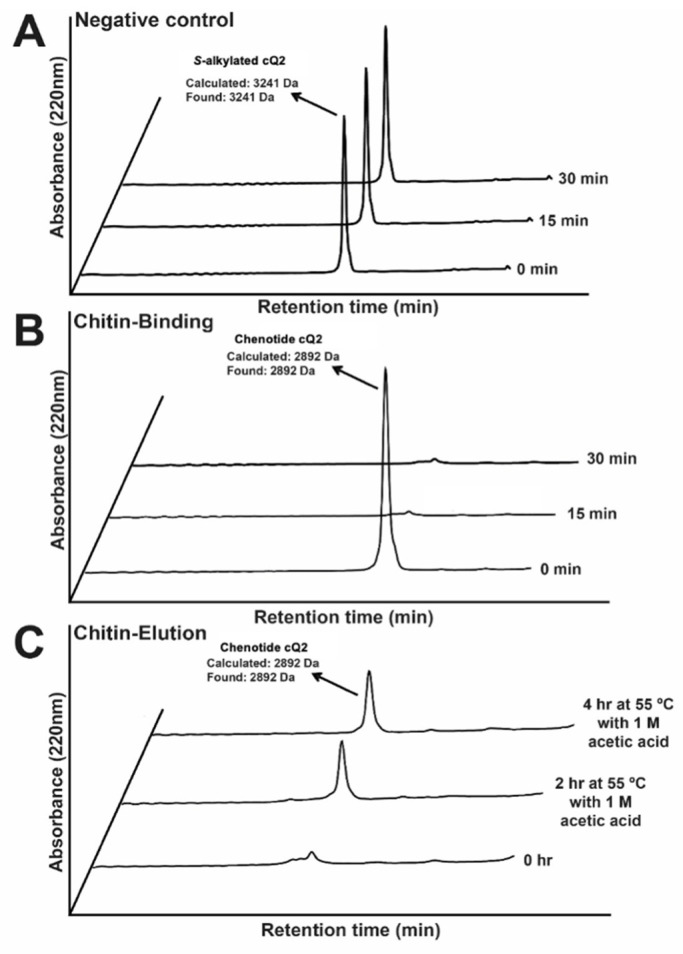
Chenotide cQ2 is chitin binding. Comparison of chitin binding activities of (**A**) chenotide cQ2 and (**B**) *S*-alkylated cQ2 (iodoacetamido-) using chitin resins. The supernatants were analyzed by RP-HPLC. (**C**) Elution profile of chenotide cQ2 from chitin resin using 1M acetic acid at 55 °C. The supernatants were analyzed by RP-HPLC.

To confirm the chitin binding activity of chenotide cQ2, the native peptides were incubated with chitin beads at 25 °C, and the *S*-alkylated forms were used as a control (Figure 5). After incubation for 1 h, analysis using RP-HPLC revealed complete depletion of the native chenotide cQ2 from the incubating solution, suggesting that this peptide binds the chitin beads. This binding was confirmed after eluting chenotide cQ2 from the chitin beads using approximately 30% 1 M acetic acid at 55 °C. On the other hand, the control *S*-alkylated chenotide cQ2 did not bind to the chitin resin, indicating that the cystine-knot disulfide scaffold is important to maintain the surface topology of the CB domain, for the recognition and binding of chitin.

### 2.7. Chenotide cQ2 Is an Anti-Fungal Peptide

**Figure 6:**
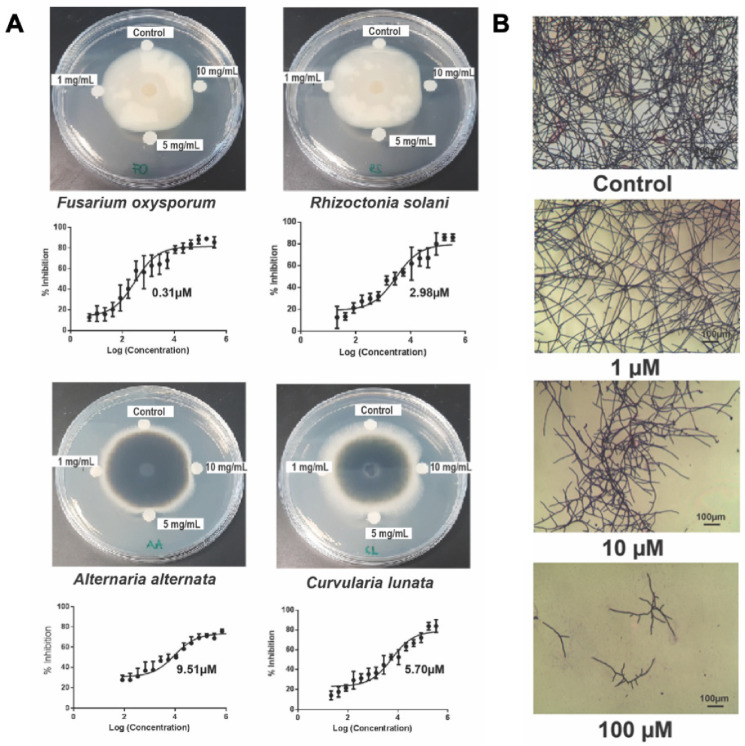
Chenotide cQ2 is an anti-fungal peptide. (**A**) Fungal inhibition of chenotide cQ2 against *Fusarium oxysporum*, *Rhizoctonia solani*, *Alternaria alternata* and *Curvularia lunata*. Formation of arc-shaped inhibition zones in the disc diffusion assay indicated susceptibility of fungi towards chenotide. The IC_50_ was calculated based on the dose-response curve obtained from the micro-broth dilution assay. (**B**) Bright-field microscopy of hyphal growth inhibition with chenotide cQ2. *Fusarium oxysporum* treated with different concentrations of chenotide cQ2. Formation of stunted hyphae ends indicated that chenotide inhibits hyphal growth at the ends of the fungal mycelia.

Our laboratory previously showed that four CB-HLPs, isolated from various plants, displayed anti-fungal activities (Vaccatides from *Vaccaria hispanica*, morintides from *Moringa oleifera*, gingkotides from *Ginkgo biloba*, and altides from *Alternanthera sessilis*) [7,19,22,23]. Using four phyto-pathogenic fungal strains of *Alternaria alternata*, *Curvularia lunata*, *Fusarium oxysporum,* and *Rhizoctonia solani,* we examined the anti-fungal activity of chenotide cQ2. The results of the disc diffusion assay showed that chenotide cQ2 was effective against all four fungal strains (Figure 6A). The microbroth dilution assay showed that the IC_50_ for chenotide cQ2 against *Alternaria alternata*, *Curvularia lunata*, *Fusarium oxysporum,* and *Rhizoctonia solani* was approximately 9, 5, 0.3, and 3 µM, respectively. To show that chenotide cQ2 inhibits hyphae growth, *Fusarium oxysporum* fungal spores were treated with either peptide. Microscopic analysis revealed that both peptides indeed stunted hyphae growth (Figure 6B).

### 2.8. Chenotide Precursors Belong to a New Family of Cleavable Hololectins

**Figure 7:**
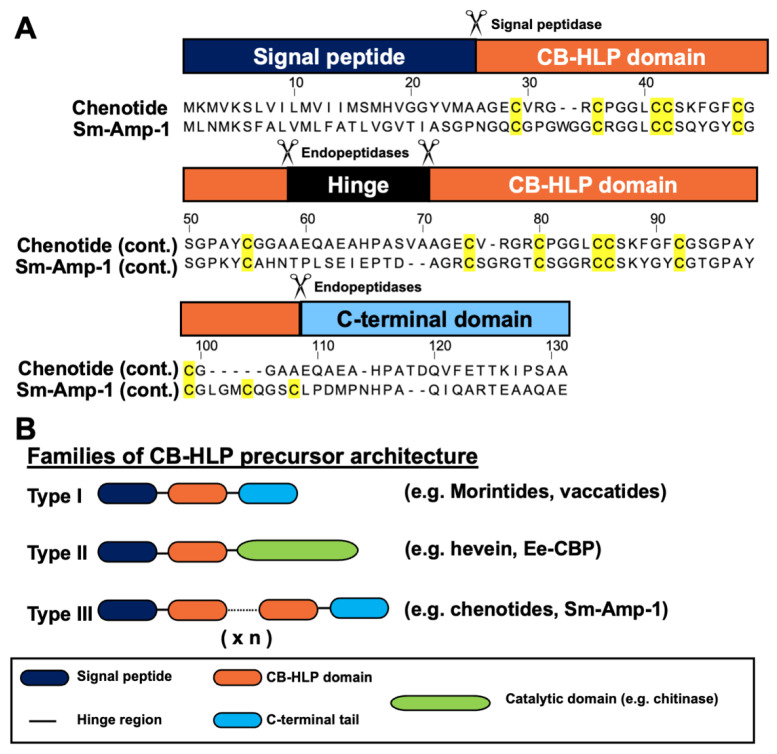
Biosynthesis pathway of chenotides. (**A**) Alignment of precursor sequence of chenotide and Sm-Amp-1 (UniProtKB-E1UYT9). Chenotide precursor contains an unusual three-domain precursor, consisting of an N-terminal signal peptide, two identical tandem-repeating mature CB-HLP domains, and a C-terminal tail. Signal peptide is cleaved by signal peptidase, C-terminal domain, and hinge region are cleaved by an alanine endopeptidase to release each mature chenotide domain. (**B**) Schematic illustration of type I (non-cargo-carrying), type II (cargo-carrying), and type III (tandem-repeating) precursor architectures of chitin-binding hevein-like peptides. x n represents number of CB-HLP domain.

Based on primary sequence determination, we showed that chenotides were biosynthesized as an unusual three-domain precursor, consisting of an N-terminal signal peptide, two tandem-repeating, identical mature CB-HLP domains, and a C-terminal tail (Figure 7A). The hinge region connecting the two mature CB-HLP domains has 18 residues and is Ala-rich. The cleavage site located between Ala-Ala suggests the involvement of cathepsin-like endopeptidases in the bioprocessing and release of the mature domains. A similar precursor architecture has been reported for another six-cysteine CB-HLP, Sm-Amp-1 (UniProtKB-E1UYT9), from chickweed (*Stellaria media*). Unlike chenotides, the Sm-Amp-1 precursor does not possess two identical mature CB-HLP domains [39].

**Figure 8:**
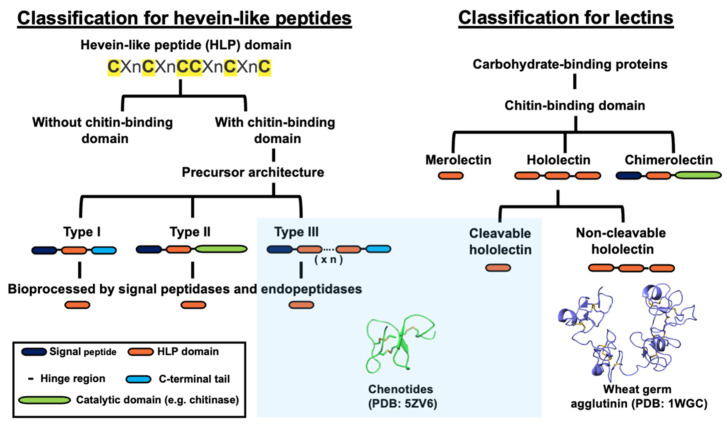
Classification of hevein-like peptides and lectins. Chenotides belongs to the family of chitin-binding hevein-like peptides with type III precursor architecture, as well as the family of cleavable hololectin. x n represents number of CB-HLP domain.

CB-HLPs are ribosomal-synthesized peptides that are processed from a common three-domain precursor architecture, consisting of a signal peptide domain, a mature peptide domain, and a C-terminal domain [7,19,22]. CB-HLPs have several subtypes of precursor architectures (Figure 7B and Figure 8) [34]. Type I has a three-domain precursor comprising a signal peptide, mature CB-HLP peptide, and a short C-terminal tail. Some examples include altides and Ar-AMP [23,40]. Type II also has three domains with a long C-terminal tail that usually encodes bioactive protein cargoes, such as proteins having a Barwin-like or class I chitinase-like domain [22,34] An example is Ee-CBP from *Euonymus europaeus,* which has a long C-terminal chitinase-like domain [41,42]. In this study, the chenotide precursors belong to a type III variant that is similar to Sm-Amp-1 [39]. Instead of a three-domain arrangement, the precursor architecture has tandem repeats of the CB-HLP domain. Chenotide precursors contain two identical repeats of the mature peptide domain, with a cleavable hinge region. This pattern was also observed in cyclotides, such as Tiptop3 from *Mormodica cochinchinensis,* which encodes eight cyclic CRPs, with potent trypsin inhibitory and insecticidal activities [43]. Thus, gene amplification could be an evolutionarily advantageous trait to boost the biosynthetic efficiency of these CB-HLPs, to benefit plant survival and reproduction [44]. 

Due to their carbohydrate-binding properties, CB-HLPs are also grouped as the following lectins: merolectins, chimerolectins, and hololectins (Figure 8) [34]. This nomenclature was assigned based on the number of carbohydrate-binding domains present in the mature peptide sequence [45,46]. Non-cargo-carrying CB-HLPs are categorized as merolectins that do not exhibit agglutinin activity [47]. Cargo-carrying CB-HLPs are categorized as chimerolectins that contain one or more carbohydrate-binding domains, linked to a long catalytic protein cargo, such as chitinase [47]. Lastly, hololectins, such as wheat germ agglutinin (WGA), are tandem-repeating carbohydrate-binding proteins with agglutin activity [48,49]. Hololectins are genetically expressed and released as a single multi-modular unit with tandem-repeating domains, connected by linkers that are commonly known as hinge regions [48,49]. 

According to lectin nomenclature, chenotide precursors belong to the group of hololectins, due to the presence of tandem repeats in their CB-HLP domain. Hololectins are expressed as a single-chain multi-modular protein, with tandem repeats of a carbohydrate-binding domain connected by hinge sequences [47]. Examples are a 171 amino acid residue WGA isolated from *Triticum vulgaris* [49], and a 227 amino acid residue *Oryza sativa* agglutinin (OSA) [50]. Unlike hololectins, the tandem repeats of chenotides were observed at the gene, but not protein level. Each modular CB-HLP domain in the chenotide precursor is released as mature CB-HLPs. Thus, we classify them under a new family of cleavable hololectins. 

**Figure 9:**
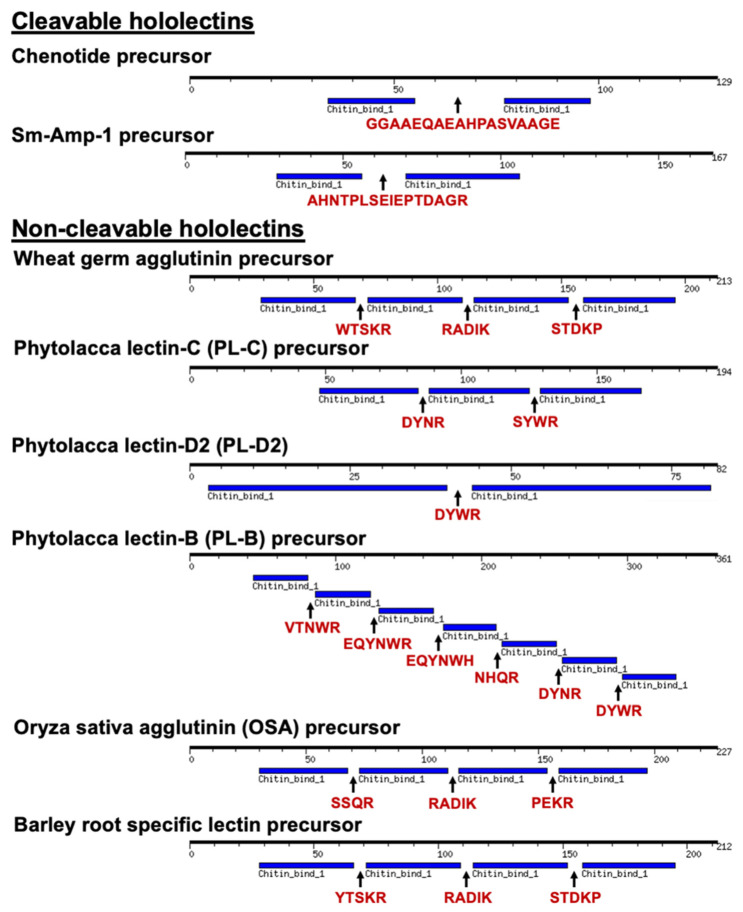
Precursor architecture of cleavable hololectins (including chenotide, and Sm-Amp-1: UniProtKB-E1UYT9) and non-cleavable hololectins (including wheat germ agglutinin (WGA: UniProtKB-P02876), phytolacca lectin-C (PL-C: UniProtKB-Q9AYP9), phytolacca lectin-D2 (PL-D2: UniProtKB-P83790), phytolacca lectin-B (PL-B: UniProtKB-Q9AVB0), Oryza sativa agglutinin (OSA: UniProtKB-Q0JF21), and barley root-specific lectin: UniProtKB-P15312). The amino acid sequences of hinge regions are colored in red. Chitin-binding hevein-like peptide domains are colored in blue. The hinge regions of non-cleavable hololectins are 4–6 amino acid residues in length, whereas the hinge region of chenotide and Sm-Amp-1 precursors are 18 and 16 amino acid residues long, respectively.

To identify the important features that differentiate precursors of cleavable hololectins from non-cleavable hololectins, we performed a sequence comparison of six well-characterized, non-cleavable hololectins from UniProt, including WGA [48,49], phytolacca lectin-C (PL-C) [51], phytolacca lectin-D2 (PL-D2) [52], phytolacca lectin-B (PL-B) [53], *Oryza sativa* agglutinin (OSA) [50], and barley root-specific lectin with chenotide [54] (Figure 9). Our findings revealed that the hinge regions of non-cleavable hololectins are highly conserved, and are 4–6 amino acid residues in length. In contrast, chenotide and Sm-Amp-1 precursors possess longer hinge regions of 16–18 amino acid residues. However, the linkers of chenotide precursors differ from the Sm-Amp-1 precursor, by being Gly/Ala-rich. As such, these linkers are susceptible to the cleavage by cathepsin-like endopeptidases. This extended spacer could facilitate the access of endopeptidases to cleave and release each CB-HLP domain. Further investigations are warranted to understand the unique characteristic of these hinge regions of the new cleavable hololectin family.

## 3. Materials and Methods

### 3.1. Materials

All chemicals and solvents, unless otherwise stated, were purchased from Sigma Aldrich, St. Louis, MO, US, and Fisher Scientific, Cleveland, OH, US.

### 3.2. Plant Materials

Different varieties of quinoa were purchased from Seedville, Canton, OH, USA. Authentication was conducted by Mr. Paul Leong at the Singapore Botany Center based on macroscopic and microscopic analyses. A voucher for each sample was deposited at the Nanyang Technological University Herbarium, School of Biological Sciences, Singapore. 

### 3.3. Extraction, Isolation, and Purification

Small-scale screening of quinoa was performed by mixing 0.1 g of sample with 1 mL water for 1 h. The crude extract was centrifuged at 9500 rpm for 10 min. The supernatant was subjected to C_18_ ZipTip and eluted with 80% ACN. For large-scale extraction, 2 kg of samples were homogenized in 20 L water for 3 h. The crude extract was centrifuged at 9500 rpm for 20 min at 4 °C. The supernatant was incubated with 80% ammonium sulfate for 1 h and centrifuged at 9500 rpm for 20 min at 4 °C. The pellet was re-suspended in 10% ACN and 1 h later, was centrifuged at 9500 rpm for 20 min at 4 °C. The filtered supernatant was loaded on a flash column packed with 500 g of C_18_ powder (Grace, Columbia, MD, US) in a Büchner funnel. Elution was performed using increasing concentrations of ethanol (20–80%). Eluents that contained the peptide of interest were pooled and purified using multiple rounds of SCX- and RP-HPLC. Fractions from SCX-HPLC containing the peptide of interest were pooled and purified by RP-HPLC.

### 3.4. Matrix-assisted Laser Desorption/Ionization Time-of-Flight Mass Spectrometry

Matrix-assisted laser desorption/ionization time-of-flight mass spectrometry (MALDI-TOF MS/MS; AB SCIEX 5800 MALDI-TOF/TOF, ABsciex, Foster City, CA, USA) was used in this study. The MALDI-MS spectra were acquired with a laser intensity of 3500, total laser shots were 2250. MALDI-TOF MS/MS spectra were acquired with a laser intensity of 5000, total laser shots were 5000.

### 3.5. Sequence Determination 

The primary sequences of the chenotides were determined by MS/MS sequencing. Peptide (40 µg) was incubated with 20 mM dithiothreitol (DTT) at 37 °C for 1 h to reduce the disulfide bonds. *S*-reduced chenotides were digested with trypsin or chymotrypsin in 5:1 (*v*/*v*) ratio in ammonium bicarbonate buffer (25 mM), pH 8 at 37 °C for 10 min. Following digestion, the digested peptides were subjected to MALDI-TOF MS/MS sequencing. Assignment of isobaric residues Lys/Gln and Leu/Ile were based on sequence comparison to genomic or expressed sequence tag (EST) data from the National Center for Biotechnology Information (NCBI) database. 

### 3.6. NMR Structural Study

All NMR experiments were conducted on a BRUKER Avance 800 NMR spectrometer (Bruker Daltonics, Bremen, Germany) with a cryogenic probe at 25 °C. The concentration of each peptide was around 1 mM in 5% D_2_O and 95% H_2_O (pH 3.5). For ^1^H, ^1^H-2D TOCSY and NOESY, the mixing times were 80 and 200 ms, respectively. The spectral width was 12 ppm for both dimensions. The NMR spectra were processed using NMRPipe software (http://spin.niddk.nih.gov/NMRPipe/) (accessed on 30 August 2021) [55]. All data analyses were performed using Sparky software (http://www.cgl.ucsf.edu/home/sparky/) (accessed on 30 August 2021) based on the 2D NOESY and TOCSY results [56]. The proton chemical shift assignments for each amino acid residue were achieved by 2D TOCSY and NOESY, while the proton–proton distance restraints were obtained from 2D NOESY by the intensities of NOE cross peaks. The chenotide solution structures were calculated using CNSsolve 1.3 software (http://cns-online.org/v1.3/) (accessed on 30 August 2021) [57]. The proton–proton distance restraints and hydrogen bonds were employed in a standard simulated annealing protocol. The distance restraints were divided into the following three classes based on NOE cross-peak intensities: strong, 1.8 < d < 2.9 Å; medium, 1.8 < d < 3.5 Å; and weak, 1.8 A < d < 5 Å. Eight hydrogen bonds were used in the structure calculation. A total of 100 structures were calculated. Twenty and 10 of the lowest-energy structures were chosen for data statistics and presentation, respectively. The structure was verified using the PROCHECK program [58] and presented using Chimera version 1.11.2 (https://www.cgl.ucsf.edu/chimera/) (accessed on 30 August 2021) [59]. 

### 3.7. Ligand Peptide Docking

Prior to ligand and peptide docking, both the peptide and ligand were prepared using the Chimera version 1.11.2 (https://www.cgl.ucsf.edu/chimera/) (accessed on 30 August 2021) for the addition of hydrogen atoms and conversion of PDB format to MOL2 format. The GOLD 5.4.0 version (Cambridge Crystallographic Data Centre, Cambridge, UK) was utilized to perform ligand–peptide docking using the NMR structure of chenotide cQ2 (PDB: 5ZV6). The GOLD score function takes into consideration the hydrogen bond and van de Waals energy. To define the active site pocket, one atom on the active site was chosen to define the pocket radius. The other settings in the program were set at default function.

### 3.8. Chitin-binding Assay

Chitin binding assays were performed as described previously [23]. *S*-alkylated and purified cQ2 were mixed with chitin beads (80 µL) (New England BioLabs, Ipswich, MA, USA) in chitin binding buffer and incubated at 25 °C for 30 min. The mixture was then washed with chitin binding buffer (50 mM Tris HCl, 500 mM NaCl, pH8) to remove unbound peptide. Elution of bound peptide was performed with 1 M acetic acid. The supernatant and eluent were analyzed using RP-UPLC and MALDI-TOF MS to assess binding and elution.

### 3.9. Peptide Stability Assays

Assessments of thermal, acidic and proteolytic stability were performed as previously described [19]. Purified chenotide cQ2 was incubated at the stated conditions and recommended buffer solution. At each time interval, samples were aliquoted in triplicate and RP-UPLC was performed. The area under the peak was used to determine the amount of chenotide present before and after treatment. 

### 3.10. Anti-Fungal Assay

A radial disc diffusion assay was used to assess chenotide anti-fungal activity. The following four phyto-pathogenic fungal strains were obtained from the China Center of Industrial Culture Collection (Beijing, China): *Alternaria alternata* (CICC 2465), *Curvularia lunata* (CICC 40301), *Fusarium oxysporum* (CICC 2532) and *Rhizoctonia solani* (CICC 40259). Fungal strains were grown on potato dextrose agar plates at 25 °C. When sufficient growth was observed, a hole was punched in the culture and the plug was transferred to a new agar plate. The plate was then incubated at 25 °C for 48 h until a radial mycelial colony formed. Paper discs with a diameter of 0.65 cm were soaked with 20 µL of peptides and then placed equidistant from the growing ends of the mycelia. Deionized water was used as a negative control. The formation of an arc-shape inhibition zone around the disk indicated anti-fungal activity.

The half-maximal inhibitory concentration levels (IC_50_) of peptides were determined by a microbroth dilution assay [60]. Fungal spores were harvested from a 4-day-old actively growing fungal plate and suspended in half-strength potato dextrose broth. Spore suspensions (1 × 10^5^ cells/mL) were mixed with peptides at varying concentrations in 96-well microplates and incubated at 25 °C for 24 h. The cells were then fixed with 100% methanol for 15 min and stained for 45 min with crystal violet dye. Excess dye was removed with MilliQ water. Elution was performed using 1:1 (*v*/*v*) ethanol/0.1 N HCl. The absorbance was measured at 570 nm.

### 3.11. Bioinformatics and Statistical Analysis

Genes encoding chenotides were obtained from the NCBI GenBank (accession number: LPWI01001647.1, NSDK01000945.1, BDCQ01007939.1) and translated using the ExPaSy translation tool (https://web.expasy.org/translate/) (accessed on 30 August 2021) [61]. The identification of the signal peptide cleavage site was determined by SignalP 4.0 (http://www.cbs.dtu.dk/services/SignalP-4.0/) (accessed on 30 August 2021) [62]. 

## 4. Conclusions

This study expands our knowledge on the occurrences and biosynthesis of CB-HLPs. We discovered, identified, and characterized novel hyperstable anti-fungal CB-HLPs chenotides from quinoa. We showed that the biosynthesis of chenotides is novel and belongs to a new cleavable hololectin family, which we have labeled as type III lectin precursor. A characteristic of such a precursor is that it contains tandem repeats of the mature peptide domains, with a cleavable Gly/Ala-rich linker consisting of 18 amino acids. Furthermore, chenotides are chitin binding and can inhibit the growth of phyto-pathogenic fungi. Taken together, the occurrence of chenotides as natural anti-microbial agents in quinoa could be the underlying reason for their extended shelf-life and unintended selection as essential staples throughout the history of humankind.

## Figures and Tables

**Table 1:** Sequence comparison of the primary peptide sequences of chenotides and reported six-cysteine chitin-binding hevein-like peptides.

Peptide	Species	Amino Acid Sequence	Mass (Da) ^1^	Charge ^2^	pI	Similarity (%)
cQ1	*C. quinoa*	AGECVRGRCPGGLCCSKFGFCGSGPAYCGGA	2963	+2	8.35	100
cQ2	*C. quinoa*	AGECVRGRCPGGLCCSKFGFCGSGPAYCGG	2892	+2	8.35	100
cQ3	*C. quinoa*	AGECVRGRCPGGLCCSKFGFCGSGPAYCG	2835	+2	8.35	100
Ac-AMP1	*A. caudatus*	VGECVRGRCPSGMCCSQFGYCGKGPKYCG	3025	+3	8.66	89.3
Ac-AMP2	*A. caudatus*	VGECVRGRCPSGMCCSQFGYCGKGPKYCGR	3181	+4	8.92	89.3
Ar-AMP	*A. retroflexus* L.	AGECVQGRCPSGMCCSQFGYCGRGPKYCGR	3153	+3	8.68	89.7
IWF4	*B.vulgaris* L.	SGECNMYGRCPPGYCCSKFGYCGVGRAYCG	3181	+2	8.27	80.0
aSG1	*A. sessilis*	APGQCNHGRCPSGLCCSQYGYCGTGPAYCG	3004	+1	7.82	89.3
aSG2	*A. sessilis*	AGECNHGRCPSGLCCSQYGYCGTGPRYCG	2992	+1	7.82	86.2
aSG3	*A. sessilis*	APGQCNHGRCPSGICCSQYGYCGTGPAYCGG	3060	+1	7.82	89.7
aSR1	*A. sessilis*	VGECVQGRCPPGLCCSRFGYCGTGPAYCG	2932	+1	7.75	96.4
aSR2	*A. sessilis*	APGECKHGRCPPGICCSQYGYCGTGPAYCG	3029	+1	7.81	89.3
aSR3	*A. sessilis*	APGECKHGRCPPGICCSQYGYCGTGPAYC	2973	+1	7.81	88.9
SmAMP1.1a	*S. media*	VGPGGECGRFGGCAGGQCCSRFGFCGSGPKYCAH	3305	+2	8.34	78.6
SmaAMP3	*S. media*	SGPNGQCGPGWGGCRGGLCCSQYGYCGSGPKYCAH	3460	+2	8.29	75.9

^1^ Mass (Da): represents the experimentally found molecular weight. ^2^ Charge: represents the total charge of the molecule, and calculated by the sum of positive (lysine, arginine and histidine residues) and negative (glutamate and aspartate residues) charges.

## Data Availability

The data presented in this study are available on request from the corresponding authors.

## Supplementary Materials

The following are available online. Supplementary figure S1. MALDI-TOF MS profile of chenotides from C. quinoa var. Willd. (A) Crude extract containing chenotides cQ1, cQ2 and cQ3; (B) S-reduction of crude extract by dithiothreitol at 37 °C for 1 h. (C) S-alkylation of crude extract by iodoacetamide at 37 °C for 1 h. Supplementary figure S2. De novo sequencing of chenotide cQ1. Tandem MALDI-TOF TOF MS/MS profiles of two trypsinized fragments of chenotide cQ1 (1695.6 *m*/*z* and 1293.4 *m*/*z*) provided the full sequence of chenotide cQ1. Supplementary figure S3. De novo sequencing of chenotide cQ2. Tandem MALDI-TOF TOF MS/MS profiles of two trypsinized fragments of chenotidecQ2 (634.3 *m*/*z* and 2283.8 *m*/*z*) provided the full sequence of chenotide cQ2. Supplementary figure S4. De novo sequencing of chenotide cQ3. Tandem MALDI-TOF TOF MS/MS profiles of two trypsinized fragments of chenotide cQ3 (634.2 *m*/*z* and 2226.7 *m*/*z*) provided the full sequence of chenotide cQ3. Supplementary Table S1. Structural statistics for the final 20 conformers of cQ2a. Supplementary Table S2. Proton chemical shift assignments for each amino acid residues of peptide cQ2.

## Sample Availability

Samples of the compounds chenotide cQ2 are available from the authors.

## References

[1] Bazile D., Jacobsen S.-E., Verniau A. (2016). The global expansion of quinoa: Trends and limits. Front. Plant Sci..

[2] Graf B.L., Rojas-Silva P., Rojo L., Delatorre-Herrera J., Baldeón M.E., Raskin I. (2015). Innovations in health value and functional food development of quinoa (*Chenopodium quinoa* Willd.). Compr. Rev. Food Sci. Food Saf..

[3] Walling L.L. (2009). Chapter 13 Adaptive defense responses to pathogens and insects. Adv. Bot. Res..

[4] Doughari J. (2015). An overview of plant immunity. J. Plant Pathol. Microbiol..

[5] Delaux P.-M., Schornack S. (2021). Plant evolution driven by interactions with symbiotic and pathogenic microbes. Science.

[6] Egorov T.A., Odintsova T.I. (2012). Defense peptides of plant immunity. Russ. J. Bioorganic Chem..

[7] Kini S.G., Wong K.H., Tan W.L., Xiao T., Tam J.P. (2017). Morintides: Cargo-free chitin-binding peptides from Moringa oleifera. BMC Plant Biol..

[8] Tam J.P., Wang S., Wong K.H., Tan W.L. (2015). Antimicrobial peptides from plants. Pharmaceuticals.

[9] Montesinos E. (2007). Antimicrobial peptides and plant disease control. FEMS Microbiol. Lett..

[10] Lenardon M.D., Munro C., Gow N.A. (2010). Chitin synthesis and fungal pathogenesis. Curr. Opin. Microbiol..

[11] Gidrol X., Chrestin H., Tan H., Kush A. (1994). Hevein, a lectin-like protein from *Hevea brasiliensis* (rubber tree) is involved in the coagulation of latex. J. Biol. Chem..

[12] Van Parijs J., Broekaert W.F., Goldstein I.J., Peumans W.J. (1991). Hevein: An antifungal protein from rubber-tree (*Hevea brasiliensis*) latex. Planta.

[13] Archer B.L. (1960). The proteins of *Hevea brasiliensis* Latex. 4. Isolation and characterization of crystalline hevein. Biochem. J..

[14] Rodriguez-Romero A., Ravichandran K., Soriano-García M. (1991). Crystal structure of hevein at 2.8 Å resolution. FEBS Lett..

[15] Andersen N.H., Cao B., Rodriguez-Romero A., Arreguin B. (1993). Hevein: NMR assignment and assessment of solution-state folding for the agglutinin-toxin motif. Biochemistry.

[16] Loo S., Kam A., Xiao T., Tam J.P. (2017). Bleogens: Cactus-Derived Anti-Candida Cysteine-Rich Peptides with Three Different Precursor Arrangements. Front. Plant Sci..

[17] Kam A., Loo S., Fan J.-S., Sze S.K., Yang D., Tam J.P. (2019). Roseltide rT7 is a disulfide-rich, anionic, and cell-penetrating peptide that inhibits proteasomal degradation. J. Biol. Chem..

[18] Loo S., Kam A., Li B.B., Feng N., Wang X., Tam J.P. (2021). Discovery of Hyperstable Noncanonical Plant-Derived Epidermal Growth Factor Receptor Agonist and Analogs. J. Med. Chem..

[19] Wong K.H., Tan W.L., Serra A., Xiao T., Sze S.K., Yang D., Tam J.P. (2016). Ginkgotides: Proline-Rich Hevein-Like Peptides from Gymnosperm Ginkgo biloba. Front. Plant Sci..

[20] Tam J.P., Nguyen G.K.T., Loo S., Wang S., Yang D., Kam A. (2018). Ginsentides: Cysteine and glycine-rich peptides from the ginseng family with unusual disulfide connectivity. Sci. Rep..

[21] Loo S., Kam A., Xiao T., Nguyen G.K.T., Liu C.F., Tam J.P. (2016). Identification and Characterization of Roseltide, a Knottin-type Neutrophil Elastase Inhibitor Derived from Hibiscus sabdariffa. Sci. rep..

[22] Wong K.H., Tan W.L., Kini S.G., Xiao T., Serra A., Sze S.K., Tam J.P. (2017). Vaccatides: Antifungal Glutamine-Rich Hevein-Like Peptides from Vaccaria hispanica. Front. Plant Sci..

[23] Kini S.G., Nguyen P.Q.T., Weissbach S., Mallagaray A., Shin J., Yoon H.S., Tam J.P. (2015). Studies on the Chitin Binding Property of Novel Cysteine-Rich Peptides from Alternanthera sessilis. Biochemistry.

[24] Li S.-S., Claeson P. (2003). Cys/Gly-rich proteins with a putative single chitin-binding domain from oat (*Avena sativa*) seeds. Phytochemistry.

[25] Lee O.S., Lee B., Park N., Koo J.C., Kim Y.H., Karigar C., Chun H.J., Jeong B.R., Kim D.H., Nam J. (2003). Pn-AMPs, the hevein-like proteins from Pharbitis nil confers disease resistance against phytopathogenic fungi in tomato, *Lycopersicum esculentum*. Phytochemistry.

[26] Koo J.C., Lee S.Y., Chun H.J., Cheong Y.H., Choi J.S., Kawabata S.-i., Miyagi M., Tsunasawa S., Ha K.S., Bae D.W. (1998). Two hevein homologs isolated from the seed of Pharbitis nil L. exhibit potent antifungal activity. Biochim. Biophys. Acta Protein Struct. Mol. Enzymol..

[27] Martins J., Maes D., Loris R., Pepermans H.A., Wyns L., Willem R., Verheyden P. (1996). 1H NMR Study of the Solution Structure of Ac-AMP2, a Sugar Binding Antimicrobial Protein Isolated fromAmaranthus caudatus. J. Mol. Biol..

[28] Broekaert W.F., Marien W., Terras F.R.G., De Bolle M.F.G., Proost P., Van Damme J., Dillen L., Claeys M., Rees S.B. (1992). Antimicrobial peptides from Amaranthus caudatus seeds with sequence homology to the cysteine/glycine-rich domain of chitin-binding proteins. Biochemistry.

[29] Huang R.-H., Xiang Y., Liu X.-Z., Zhang Y., Hu Z., Wang D.-C. (2002). Two novel antifungal peptides distinct with a five-disulfide motif from the bark of Eucommia ulmoides Oliv. FEBS Lett..

[30] Huang R.-H., Xiang Y., Tu G.-Z., Zhang Y., Wang D.-C. (2004). Solution structure of Eucommia antifungal peptide: A novel structural model distinct with a five-disulfide motif. Biochemistry.

[31] Xiang Y., Huang R.-H., Liu X.-Z., Zhang Y., Wang D.-C. (2004). Crystal structure of a novel antifungal protein distinct with five disulfide bridges from Eucommia ulmoides Oliver at an atomic resolution. J. Struct. Biol..

[32] Silverstein K.A., Moskal Jr W.A., Wu H.C., Underwood B.A., Graham M.A., Town C.D., VandenBosch K.A. (2007). Small cysteine-rich peptides resembling antimicrobial peptides have been under-predicted in plants. Plant J..

[33] Slavokhotova A.A., Shelenkov A., Andreev Y.A., Odintsova T.I. (2017). Hevein-like antimicrobial peptides of plants. Biochem..

[34] Porto W.F., Souza V.A., Nolasco D.O., Franco O.L. (2012). In silico identification of novel hevein-like peptide precursors. Peptides.

[35] Kam A., Loo S., Dutta B., Sze S.K., Tam J.P. (2019). Plant-derived mitochondria-targeting cysteine-rich peptide modulates cellular bioenergetics. J. Biol. Chem..

[36] Verheyden P., Jurgen P., Dominique M., Henri A.M.P., Lode W., Rudolph W., JoséC M. (1995). 1H NMR study of the interaction of N, N′, N ″-triacetyl chitotriose with Ac-AMP2, a sugar binding antimicrobial protein isolated from *Amaranthus caudatus*. FEBS Lett..

[37] Andreev Y.A., Korostyleva T.V., Slavokhotova A.A., Rogozhin E.A., Utkina L.L., Vassilevski A.A., Grishin E.V., Egorov T.A., Odintsova T.I. (2012). Genes encoding hevein-like defense peptides in wheat: Distribution, evolution, and role in stress response. Biochimie.

[38] Odintsova T.I., Vassilevski A., Slavokhotova A.A., Musolyamov A.K., Finkina E.I., Khadeeva N.V., Rogozhin E.A., Korostyleva T.V., Pukhalsky V.A., Grishin E.V. (2009). A novel antifungal hevein-type peptide from Triticum kiharae seeds with a unique 10-cysteine motif. FEBS J..

[39] Slavokhotova A.A., Shelenkov A.A., Korostyleva T.V., Rogozhin E.A., Melnikova N.V., Kudryavtseva A.V., Odintsova T.I. (2017). Defense peptide repertoire of Stellaria media predicted by high throughput next generation sequencing. Biochimie.

[40] Lipkin A., Anisimova V., Nikonorova A., Babakov A., Krause E., Bienert M., Grishin E., Egorov T. (2005). An antimicrobial peptide Ar-AMP from amaranth (*Amaranthus retroflexus* L.) seeds. Phytochemistry.

[41] Bergh K.P.V.D., Van Damme E.J., Peumans W.J., Coosemans J. (2002). Ee-CBP, a hevein-type antimicrobial peptide from bark of the spindle tree (*Euonymus europaeus* L.). Mededelingen.

[42] Bergh K.P.V.D., Proost P., Van Damme J., Coosemans J., Van Damme E.J., Peumans W.J. (2002). Five disulfide bridges stabilize a hevein-type antimicrobial peptide from the bark of spindle tree (*Euonymus europaeus* L.). FEBS Lett..

[43] Craik D.J., Malik U. (2013). Cyclotide biosynthesis. Curr. Opin. Chem. Biol..

[44] Panchy N., Lehti-Shiu M., Shiu S.-H. (2016). Evolution of Gene Duplication in Plants. Plant physiol..

[45] Peumans W.J., Van Damme E. (1995). Lectins as plant defense proteins. Plant physiol..

[46] Van Damme E.J., Lannoo N., Peumans W.J. (2008). Plant lectins. Adv. Bot. Res..

[47] Mishra A., Behura A., Mawatwal S., Kumar A., Naik L., Mohanty S.S., Manna D., Dokania P., Mishra A., Patra S.K. (2019). Structure-function and application of plant lectins in disease biology and immunity. Food Chem. Toxicol..

[48] Smith J.J., Raikhel N.V. (1989). Nucleotide sequences of cDNA clones encoding wheat germ agglutinin isolectins A and D. Plant Mol. Biol..

[49] Wright H.T., Brooks D.M., Wright C.S. (1985). Evolution of the multidomain protein wheat germ agglutinin. J. Mol. Evol..

[50] Zhang W., Peumans W.J., Barre A., Astoul C.H., Rovira P., Rougé P., Proost P., Truffa-Bachi P., Jalali A.A., Van Damme E.J. (2000). Isolation and characterization of a jacalin-related mannose-binding lectin from salt-stressed rice (*Oryza sativa*) plants. Planta.

[51] Yamaguchi K.-i., Mori A., Funatsu G. (1995). The complete amino acid sequence of lectin-C from the roots of pokeweed (*Phytolacca americana*). Biosci. Biotechnol. Biochem..

[52] Yamaguchi K.-i., Mori A., Funatsu G. (1996). Amino acid sequence and some properties of lectin-D from the roots of pokeweed (*Phytolacca americana*). Biosci. Biotechnol. Biochem..

[53] Yamaguchi K.-i., Yurino N., Kino M., Ishiguro M., Funatsu G. (1997). The amino acid sequence of mitogenic lectin-B from the roots of pokeweed (*Phytolacca americana*). Biosci. Biotechnol. Biochem..

[54] Lerner D.R., Raikhel N.V. (1989). Cloning and characterization of root-specific barley lectin. Plant physiol..

[55] Delaglio F., Grzesiek S., Vuister G.W., Zhu G., Pfeifer J., Bax A. (1995). NMRPipe: A multidimensional spectral processing system based on UNIX pipes. J. Biomol. NMR.

[56] Lee W., Tonelli M., Markley J.L. (2014). NMRFAM-SPARKY: Enhanced software for biomolecular NMR spectroscopy. Bioinformatics.

[57] Brünger A.T., Adams P.D., Clore G.M., DeLano W.L., Gros P., Grosse-Kunstleve R.W., Jiang J.-S., Kuszewski J., Nilges M., Pannu N.S. (1998). Crystallography & NMR system: A new software suite for macromolecular structure determination. Acta Crystallogr. D..

[58] Laskowski R.A., Rullmann J.A.C., MacArthur M.W., Kaptein R., Thornton J.M. (1996). AQUA and PROCHECK-NMR: Programs for checking the quality of protein structures solved by NMR. J. Biomol. NMR.

[59] Huang C.C., Couch G.S., Pettersen E.F., Ferrin T.E. Chimera: An extensible molecular modeling application constructed using standard components. Proceedings of the Pacific Symposium on Biocomputing.

[60] Wiegand I., Hilpert K., Hancock R.E. (2008). Agar and broth dilution methods to determine the minimal inhibitory concentration (MIC) of antimicrobial substances. Nat. Protoc..

[61] Gasteiger E., Gattiker A., Hoogland C., Ivanyi I., Appel R.D., Bairoch A. (2003). ExPASy: The proteomics server for in-depth protein knowledge and analysis. Nucleic Acids Res..

[62] Petersen T.N., Brunak S., von Heijne G., Nielsen H. (2011). SignalP 4.0: Discriminating signal peptides from transmembrane regions. Nat. Methods.

