# Supplementary material for: Anti-Fungal Hevein-like Peptides Biosynthesized from Quinoa Cleavable Hololectins
Source: Molecules. 2021 Sep 29;26(19):5909. doi: 10.3390/molecules26195909 (PMC8512870; doi:10.3390/molecules26195909)
Supplement: Supplementary file 1 [file molecules-26-05909-s001.zip › Supplementary figues.pdf]

Supplementary figures

Anti-fungal hevein-like peptides biosynthesized from Quinoa cleavable-hololectins

Shining Loo [1,#], Stephanie V. Tay [1,#], Antony Kam [1], Fan Tang [1], Jing-Song Fan [2], Daiwen Yang [2], James P. Tam [1,*]

[1] School of Biological Sciences, Nanyang Technological University, Singapore 637551.
[2] Department of Biological Sciences, National University of Singapore, Singapore 117543.
[#] These authors contributed equally to this work

*Corresponding author: Professor James P. Tam, School of Biological Sciences, Nanyang Technological University, 60 Nanyang Drive, 637551, Singapore

Email: JPTam@ntu.edu.sg

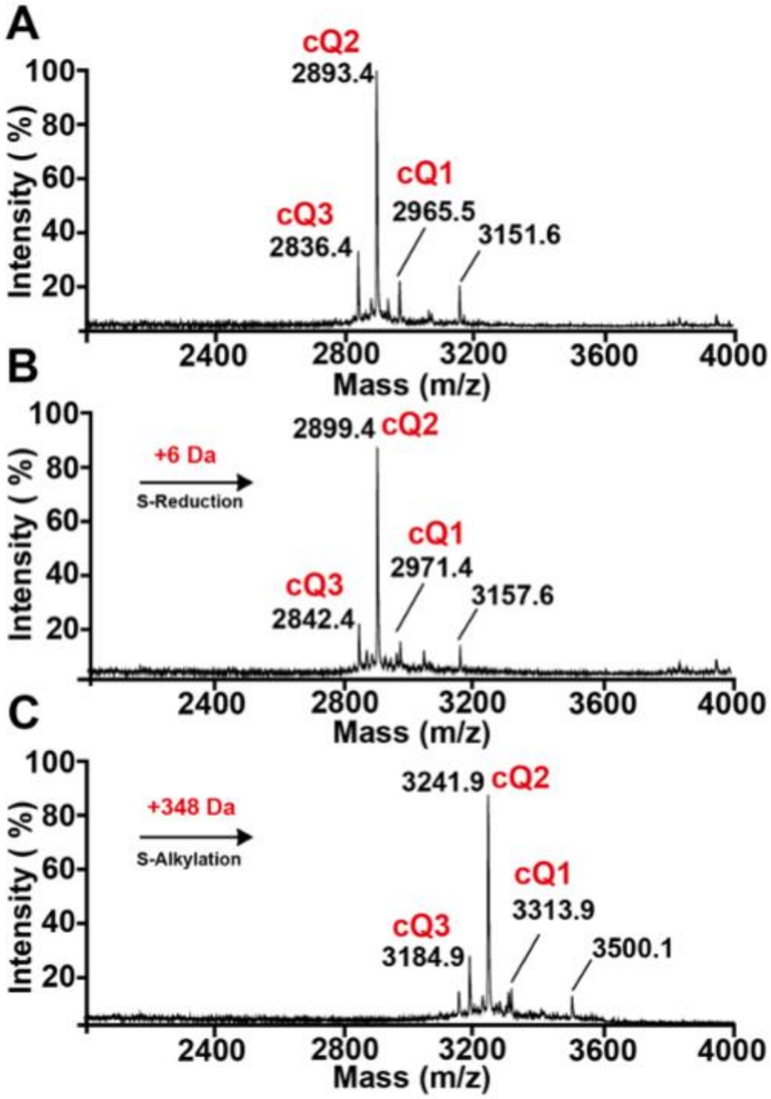

Supplementary figure S1. MALDI-TOF MS profile of chenotides from *C. quinoa* var. Willd. (A) Crude extract containing chenotides cQ1, cQ2 and cQ3; **(B)** *S*-reduction of crude extract by dithiothreitol at 37ºC for 1 h. **(C)** *S*-alkylation of crude extract by iodoacetamide at 37ºC for 1 h.

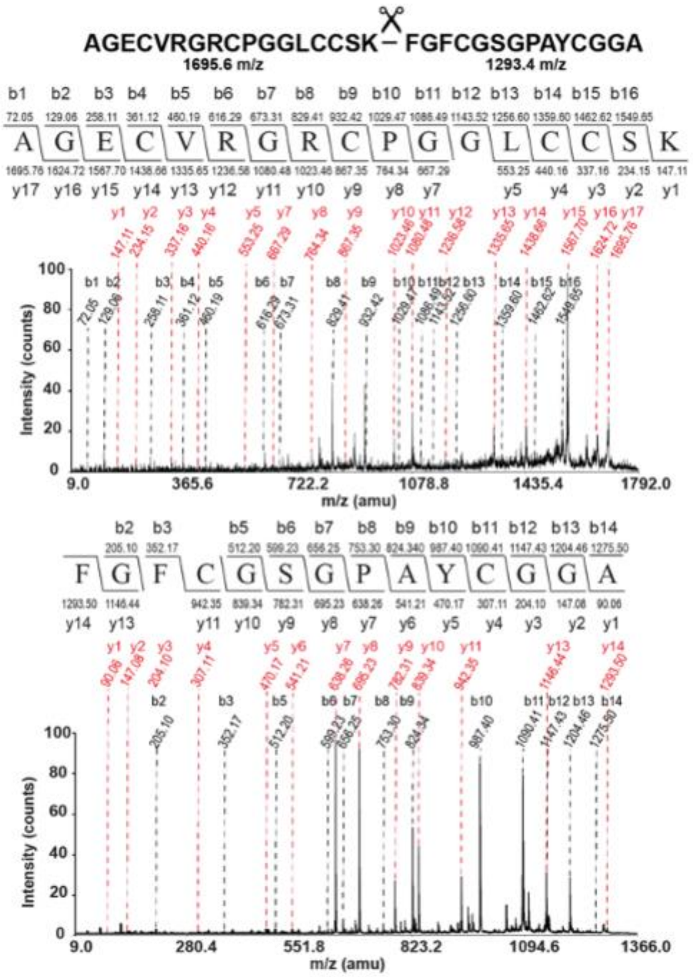

Supplementary figure S2. *De novo* sequencing of chenotide cQ1. Tandem MALDI-TOF TOF MS/MS profiles of two trypsinized fragments of chenotide cQ1 (1695.6 m/z and 1293.4 m/z) provided the full sequence of chenotide cQ1.

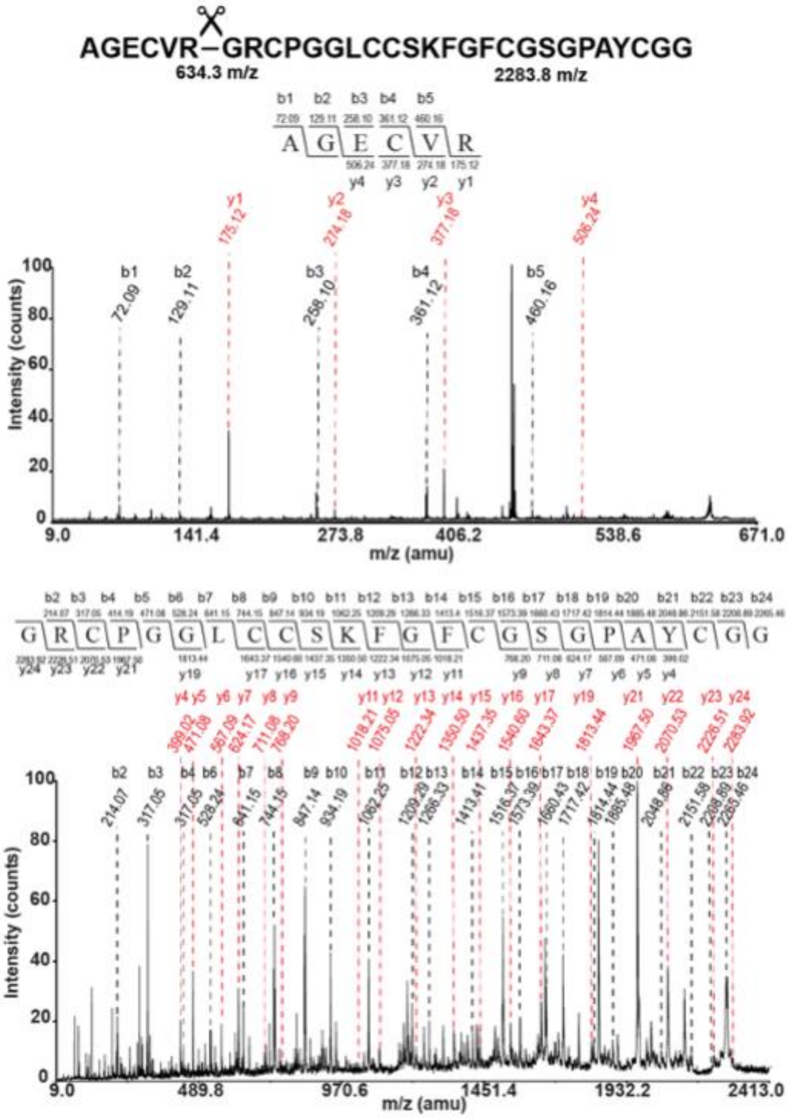

Supplementary figure S3. ***De novo*** **sequencing of chenotide cQ2**. Tandem MALDI-TOF TOF MS/MS profiles of two trypsinized fragments of chenotidecQ2 (634.3 m/z and 2283.8 m/z) provided the full sequence of chenotide cQ2.

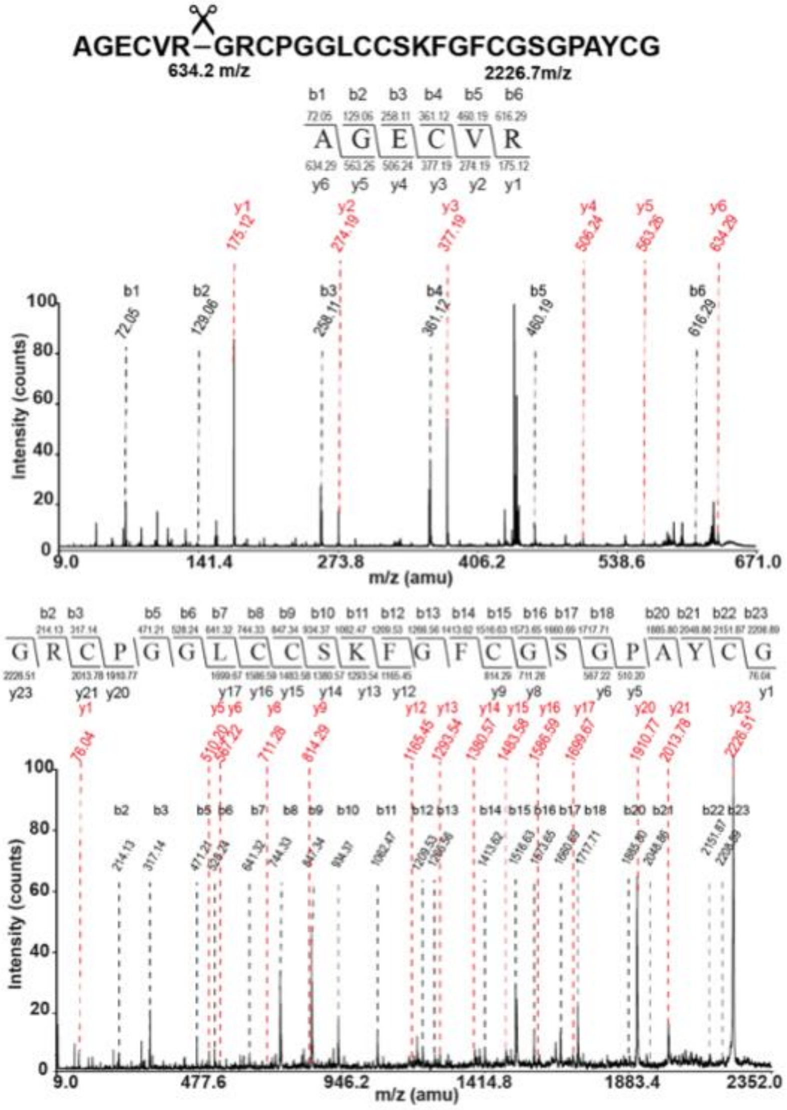

Supplementary figure S4. *De novo* sequencing of chenotide cQ3. Tandem MALDI-TOF TOF MS/MS profiles of two trypsinized fragments of chenotide cQ3 (634.2 m/z and 2226.7 m/z) provided the full sequence of chenotide cQ3.
