# Supplementary material for: Anti-Fungal Hevein-like Peptides Biosynthesized from Quinoa Cleavable Hololectins
Source: Molecules. 2021 Sep 29;26(19):5909. doi: 10.3390/molecules26195909 (PMC8512870; doi:10.3390/molecules26195909)
Supplement: Supplementary file 1 [file molecules-26-05909-s001.zip › Supplementary tables.pdf]

Supplementary tables

Anti-fungal hevein-like peptides biosynthesized from Quinoa cleavable-hololectins

Shining Loo [1,#], Stephanie V. Tay [1,#], Antony Kam [1], Fan Tang [1], Jing-Song Fan [2], Daiwen Yang [2], James P. Tam [1,*]

[1] School of Biological Sciences, Nanyang Technological University, Singapore 637551.

[2] Department of Biological Sciences, National University of Singapore, Singapore 117543.

[#] These authors contributed equally to this work

*Corresponding author: Professor James P. Tam, School of Biological Sciences, Nanyang Technological University, 60 Nanyang Drive, 637551, Singapore

Email: JPTam@ntu.edu.sg

Supplementary Table S1. Structural statistics for the final 20 conformers of cQ2[a]

Distance restraints	
Intra-residue (*i*-*j* = 0)	70
Sequential (|*i*-*j*| = 1)	68
Medium range (2 ≤ |*i*-*j*| ≤ 4)	13
Long range (|*i*-*j*| ≥ 5)	28
Hydrogen bond	8
Total	187
Average rmsd to the mean structure (Å)[b]	
Backbone atoms	0.58 ± 0.24
Heavy atoms	1.21 ± 0.27
ϕ/ψ space[c]	
Most favored region (%)	81.4
Additionally allowed region (%)	18.6
Generously allowed region (%)	0.0
Disallowed region (%)	0.0
rmsd from covalent geometry	
Bonds (Å)	0.0061 ± 0.0002
Angles (deg.)	0.540 ± 0.0319
Impropers (deg.)	0.307 ± 0.021
rmsd from experimental restraints	
NOEs (Å)	0.023 ± 0.0069

[a] Selected from 100 calculated conformers according to overall energy.
[b] Calculated with MOLMOL using range 3-9, 13-28.
[c] Calculated with PROCHECK-NMR.

Supplementary Table S2. Proton chemical shift assignments for each amino acid residues of peptide cQ2.

	HN (ppm)	Hα (ppm)	Hβ (ppm)		Others (ppm)
A1		4.170	1.562		
G2	8.889	4.018, 4.139			
E3	8.503	4.462	1.898	2.048	Hγ, 2.288, 2.335
C4	7.714	4.373	2.792	3.143	
V5	8.620	4.117	1.892		Mγ, 0.863, 0.880
R6	9.474	3.838	1.582		Hγ, 1.811, 1.936
G7	8.349	3.732, 4.080			
R8	7.820	4.713	1.817	1.886	Hγ, 1.572, 1650
C9	8.708	5.141	2.485	2.791	
P10		4.603	2.294		Hγ, 1.875; Hδ, 3.886, 3.520
G11	8.492	3.757, 3.824			
G12	8.679	3.758, 4.043			
L13	7.433	4.351	1.316	1.577	Hγ, 1.701; Mδ, 0.602, 0.699
C14	9.074	4.480	2.559	3.767	
C15	8.751	4.808	2.809	2.926	
S16	9.848	4.903	4.293	4.348	
K17	8.981	4.043	1.494	1.572	Hγ, 1.096; Hδ, 0.741, 1.106
F18	7.434	4.716	2.890	3.603	Hδ, 7.1075; He, 7.360
G19	7.954	3.561, 3.952			
F20	7.524	5.195	3.164	2.586	Hδ, 7.075; He, 7.357
C21	8.819	5.609	2.667	2.820	
G22	8.798	3.588, 1.938			
S23	8.025	5.116	3.698	3.776	
G24	8.322	3.976, 4.560			
P25		4.258	2.099		Hδ, 3.812
A26	8.759	4.125	1.293		
Y27	7.671	4.125	2.473	2.855	Hδ, 7.215; He, 6.710
C28	8.528	4.566	2.768	3.285	
G29	8.008	3.959, 4.085			
G30	8.075	3.889			
